# Why have Advance Directives failed in Spain?

**DOI:** 10.1186/s12910-020-00557-4

**Published:** 2020-11-16

**Authors:** Benjamín Herreros, María Benito, Pablo Gella, Emanuele Valenti, Beatriz Sánchez, Tayra Velasco

**Affiliations:** 1 Instituto de Ética Clínica Francisco Vallés, Universidad Europea de Madrid, Madrid, Spain; 2 Unidad de Medicina Interna, Hospital Universitario Fundación Alcorcón, Calle Budapest 1, 28922 Alcorcón, Madrid, Spain; 3 Legal Medicine, Psychiatry and Pathology Department, Universidad Complutense , Plaza de Ramón y Cajal, s/n, 28040 Madrid, Spain; 4 Instituto de Ética Clínica Francisco Vallés, Universidad Europea, Plaza de Francisco Morano, s/n, 28005 Madrid, Spain; 5 Centre for Ethics in Medicine, Bristol Medical School, University of Bristol, 39 Whatley Road, Bristol, BS8 2PS UK; 6 Facultad de Enfermería, Fisioterapia y Podología, Universidad Complutense, Plaza de Ramón y Cajal, s/n, 28040 Madrid, Spain

**Keywords:** Advance directive, Living will, Advance care planning

## Abstract

**Background:**

In Spain, there has been great effort by lawmakers to put Advance Directives (ADs) into practice since 2002. At the same time, the field of bioethics has been on the rise, a discipline that has spurred debate on the right of patients to exercise their autonomy. Despite all this, the implementation of ADs can be said to have failed in Spain, because its prevalence is very low, there is a great lack of knowledge about them and they have very little impact on clinical decisions. The purpose of this article is to analyze and discuss the main reasons for the failure of ADs in Spain.

**Main body:**

The main reasons why ADs have no impact on clinical practice in Spain have been fundamentally four: (1) the training of health professionals about the end of life and AD is lacking; (2) there has been no public process to increase awareness about AD, and therefore people (with the exception of specific highly sensitized groups) know little about them; (3) the bureaucratic procedure to document and implement ADs is excessively complex and cumbersome, creating a significant barrier to their application; (4) in Spain, the remnants of a paternalistic medical culture continue to exist, which causes shared decision-making to be difficult.

**Conclusion:**

Due to the four reasons mentioned above, AD have not been a useful tool to help honor patients’ autonomous decisions about their future care and, therefore, they have not achieved their objective. However, despite the difficulties and problems identified, it has also been observed that health care professionals and the Spanish public have a very positive view of AD. Having identified the problems which have kept AD from being successful, strategies must be developed to help improve their implementation into the future.

## Background

In Spain, there has been a great deal of effort by lawmakers to put Advance Directives (ADs) into practice since 2002. Because powers over health care in Spain have been devolved to the “Autonomous Communities” (the name for Spain’s regions), the management and implementation of ADs have been the responsibility of those Autonomous Communities [1, 2]. However, despite the fact that specific legislation has been enacted on ADs over the last 20 years, at both the national level and in the Autonomous Communities, the number of people who express their wishes about their future care through ADs is very low. In 2020, for example, only 0.6% of the Spanish population had filled out and registered the ADs document [3]. The result of this very low number of people documenting ADs is obvious: their use in and impact on clinical practice are very low.

The emerging use of ADs in Spain has taken place at the same pace as the development of bioethics, a discipline almost unheard of in Spain in the 1990s [4]. Bioethics has promoted the discussion over patients’ rights in Spain, and more specifically the right to exercise patient autonomy. This discussion and the implementation of specific laws governing ADs have led to a significant number of studies about ADs in Spain. To perform the debate on ADs in Spain, we carried out a bibliographic search on ADs in Spain. Those articles that explored the implementation of ADs in Spain (especially the knowledge about them, their prevalence and impact on clinical decisions) were selected. Our study and the article do not follow the methodology of systematic reviews, instead the bibliographic search was used to establish the debate. Some of the articles found have focused on patients (Tables 1, 2, 3) [5, 6], and others on their relatives and representatives (Table 4) [7], while there has also been a significant number of studies exploring the role played by health care professionals (Table 5) [8–11]. All of them show that, despite the positive attitude towards ADs in Spain, the Spanish people possess insufficient knowledge about them, including both professionals and the public, and their use in clinical practice is very infrequent.

**Table 1:** Studies on patients with ADs by type of care

Field	Year	Author and type of study	Results
Primary care	2000	Flordelís, Fernando Qualitative study	70 participants: ADs would be helpful in communications about end-of-life care between doctors and patients
	2003	Santos de Unamuno, Carmen Observational	107 participants. 97% of patients expressed interest in ADs, 39.3% stated that they would complete such a document, 88.8% that they would discuss it with their relatives and 73.8% with their family doctor
	2008	Ángel- López Rey, Esther Observational	395 participants. 88.8% were unfamiliar with ADs. After informing them about the document, 67.8% would fill it out if terminally ill, while 56.3% would sign it immediately. 34.4% would assign a relative to be a representative. 76.9% would keep the document once signed, while 49.3% would modify it at request of their relatives and 46.6% at request of their doctor. 85.4% would feel reassured to know that their wishes would be honored if they were to become incapacitated
	2008	Angora, Francisco Intervention study	51 participants. 47% were already familiar with ADs, and 39% knew that such a document could be completed in their region. 69% would prefer to submit the document to a public register, 12% to witnesses and 6% to the notary public. 82% would notify their family doctor. 78% believed that ADs are only taken into consideration in extreme cases
	2011	Navarro, Beatriz Qualitative study	15 participants. Positive attitude towards ADs, but great ignorance about the document itself and its purpose. Any information was mainly obtained from the media. What they had heard about ADs, they related to euthanasia and organ donation. They pointed out the need to include the document in the patient’s medical record and that the initiative to increase awareness about ADs should come from doctors themselves
	2012	Andrés-Pretel, Fernando Observational	464 participants. 86.2% of the patients were unfamiliar with them, while 3.4% had registered ADs. 76.7% showed a favorable attitude, stating that it would be advisable to register ADs. 88.2% pointed out the need to raise awareness among the entire population about the possibility of registering ADs, especially the elderly. 70.2% believed that it facilitates decision-making for the doctor and family
	2014	Llordés, Montserrat Observational	579 participants. 38% were familiar with them, while 2% had registered ADs, mostly before a notary public (62%). 74% expressed interest in receiving information, preferably in writing
	2015	Serrano, Reyes Observational	192 participants. 51% were familiar with ADs. Of them, up to 15.3% had been informed by health care professionals (16.3% by relatives and 43.9% through the media). 90.6% believed that ADs were useful, with 65.6% willing to make use of them. 60.4% believed that family doctors should offer all of their patients the chance to register ADs. 75.5% would recommend their relatives to prepare ADs
	2018	Ortiz-Gonçalves, Belén Observational	425 participants. 50% were familiar with ADs and 4% had drafted them. 63% were willing to prepare them, 45% would inform their family, and 70% would tell their doctor. 91% would like to decide about the care they receive at the end of life
Nursing home	2002	Martínez, Esther Intervention study	20 participants. 35% had their will prepared, and 60% would choose a relative as their representative. 80% had some written statement about future care, most preferring to die in the nursing home. 60% expressed the desire to receive information in the event of terminal illness or a degenerative disease
Intensive care	2003	Solsona, José F Observational	80 participants. 12.5% had verbally designated a representative. None had done so in writing. None had knowledge of living wills, nor had any formalized one
Emergencies Emergencies	2007	Antolín, Albert Observational	160 participants. 19% were familiar with ADs, and 3% had been informed about them by their doctor. 85% would designate a relative to be their representative. 50% were in favor of completing ADs after being properly informed, and 91% were in favor of distributing informative brochures
	2010	Antolín, Albert Observational	190 participants: 16% were familiar with ADs, and 5% had been informed about them by their doctor. 81% would designate a relative to be their representative. 46% were in favor of completing ADs after being properly informed, and 97% were in favor of distributing informative brochures
Internal medicine	2011	Molina, Julia Observational	155 participants. 4.5% knew what ADs were, but only 1 patient had completed ADs (before a notary public). After learning about their existence, up to 31.6% would like to prepare ADs. 80.6% were in favor of having the document in the patient’s medical record, while 72.9% did not believe that having ADs would change the doctor’s decisions. None remembered that there was a section about ADs in the hospital’s intake handbook
	2013	Pérez, María Observational	206 participants. 5.3% knew what ADs were, but only 1 patient had completed ADs. After being informed about them, up to 46.1% would like to do so. Patients remarked that having ADs would not change the doctor’s mind. Of the patients who were familiar with ADs, most knew about them through the media. 80.1% wanted the information to be included in the patient’s medical record
Palliative care	2011	Domínguez, Carmen Observational	267 participants. 11.2% knew of their existence, while 40% wished to be given further information, 62% of them being non-cancer patients. 2.25% had completed ADs. Among these, ADs had been executed before witnesses, and private documents were used; none had done so using the official form from their Autonomous Community. A legal representative was not assigned either. 100% of ADs were recorded in the patient’s medical record

**Table 2:** Studies on patients with ADs by pathology

Pathology	Year	Author and type of study	Results
HIV	2006	Miró, Gloria Observational	222 participants. 31.3% were familiar with ADs. A higher level of knowledge was found among women, individuals with a higher level of education and those who most wanted to participate in decision-making. 61.3% were of the opinion that they should be the person to decide about their own medical care. 57.2% would like to have recorded those decisions, 92.3% to designate a representative, and 70.2% to name a relative to make decisions for them
Heart failure	2010	Antolín, Albert Observational	309 participants. 13.3% were familiar with ADs and what they consisted of. Of these, up to 4.9% got this information from their doctor, 28.8% were in favor of completing ADs in the future, and 81.9% would designate a relative to be their representative. Being objectively well-informed was related with having been admitted to the ICU, having participated in decision-making and being willing to prepare ADs
Kidney failure in dialysis	2009	Palero, Claudia Observational	95 participants. 63.2% are not familiar with ADs, and 2.2% had them prepared. 5.7% have full confidence in the document, and 68.5% have full confidence in their family and doctors. They preferred human relations over documents (planning and better flowing communication). Patients trust professionals, but communication about how to face death was made difficult by taboos regarding the issue
	2011	Sánchez, José A Observational	154 participants. 7.9% had their ADs prepared, and 6.6% had designated a legal representative. 65% rejected mechanical ventilation and nasogastric tube feeding. Many patients believed that ADs should be prepared before starting dialysis treatment, though most of them pointed out that this should only be offered to those who request it. More than a half expressed that they hoped to prepare ADs
	2017	Rodríguez, Ángel Intervention study	210 participants. 41.3% stated that they wanted to limit therapeutic efforts in the severe situations found with ADs; 6.1% said they wished to continue dialysis under all circumstances; 14.7% had expressed their wishes to their representative, but without written confirmation, and 37.9% refused to complete ADs

**Table 3:** Profile of patients who complete ADs

Scope	Year	Author and type of study	Results
Valencia advance directives register	2010	Nebot, Cristina Observational	931 participants. 1.6% had registered ADs, 68.4% of these being women at an average age of 54 years. 73.8% used a document previously drawn up by a specific religious confession. 89.7% did so before witnesses and 10.3% before a notary. 95.2% designated a representative. 74.1% recorded their refusal to receive some form of treatment. Of those who did not have a declared religious confession, the main reasons for preparing ADs were: 99.2% to limit therapeutic effort and 98.4% to be administered medication to alleviate pain. 51.6% wished to donate for transplants, 16% wanted to die at home and 23% in the hospital
Advance directives register of the provincial health delegation of albacete	2014	Del Pozo, Katia Observational	123 participants. 64.2% were women; the average age was 53.3 years; 61% had completed secondary schooling, 61.8% were married and 67.5% living with a partner and/or children. Most were independent in performing their everyday activities (98.4% for basic and 94.3% for instrumental activities). 73.2% presented some sort of chronic illness. Despite having had long-lasting relationships with their doctors (9.4 years on average), conversations regarding the end of life were scarce (18.3%), though 90.1% had discussed the topic with their relatives. 54.5% had a family member who had previously formalised an ADs document, 68.5% considered it useful in the event of a loved one’s death, and 56.7% had worked as a caregiver for a terminally ill person
Customer Service at a third-tier hospital in Barcelona	2014	Antolín, Albert Observational	130 participants. 61.5% women, average age of 61 years, 64% with neoplastic disease and 33% with chronic illness. 18% presented no relevant disease. 73% were totally independent, and 36.4% had no comorbidity. 28% died while the study was carried out, and 35.1% of them presented inability to make a decision during the terminal stage of their disease, while 69% made express reference to and use of ADs in the final stage of their disease
Advance directives register of Catalonia	2016	Busquets, Josep M Observational	146 participants. 61.3% were women, and 65.1% were over the age of 70 years. 70.5% executed ADs before witnesses, and 29.5% before notaries. 25.3% refused blood transfusions, 11% donated their bodies to science, and 4.8% donated their organs

**Table 4:** Studies on ADs with patients’ relatives and representatives

Field	Year	Author and type of study	Results
Intensive care	2003	Solsona, José F Observational	80 participants. 32.5% knew the patient’s wishes, 65% of which would make the decision to limit treatment if the patient were to become seriously ill. Most were unaware of the patient’s wishes regarding organ donation, and no legal representatives had been assigned. 12.5% had been assigned but only verbally
	2010	Arauzo, Vanessa Observational	210 participants. 5% had prepared ADs, and 21% had considered preparing them. 85% expressed an interest in receiving information, and 51% believed that having a relative or a friend admitted to the ICU had caused them to reflect on this topic
Emergencies	2010	Antolín, Albert Observational	190 participants: 76% of the companions were women, generally younger than the patient, with a better knowledge of the disease (88% vs. 74%) and more ADs (28% vs. 16%) than the patients themselves
Representatives (register)	2016	Busquets, Josep M Observational	146 participants. 67.1% stated that the ADs were consulted and 58.9% that representatives were consulted, while 82.1% believed that patient’s will was respected. 69.9% believed that patients who had previously planned their care using ADs had had a good death, 22.4% stated that it could have been better, and 6.8% believed they suffered a great deal
Dialysis	2017	Rodríguez Ángel, Intervetion	76 participants. 94.7% expressed an extremely high degree of satisfaction with ADs, noting their usefulness in making decisions to limit life support treatment in situations for which the patient had previously stated his or her wishes

**Table 5:** Studies on ADs with health professionals

Field	Year	Author and type of study	Results
Primary care (doctors)	2007	Santos, Carmen Observational	169 participants. 97% considered the living will useful, though 83.2% reported not having enough information to help their patients prepare it. 95.2% agreed to address the issue of living wills, but only at the patient’s request, 72.1% in the event of chronic illness and 57.2% systematically during doctor’s visits. The main difficulties identified by doctors in the formalization of ADs were: 84.9% found legal problems between the patient’s request and the current legislation, and 80.1% discrepancies between patients’ instructions and those given by their relatives. Doctors’ information sources: 66.3% from non-health related media and 59.2% from the medical press. Most were unaware of current laws and did not how to access the registered document
Primary care (doctors)	2011	Navarro, Beatriz Qualitative study	13 participants. Overall, there was a positive attitude towards ADs, but also great ignorance about the document and its purpose. Lack of time was one of the impediments to implementing ADs during doctor’s visits. Alternatives to primary care visits are proposed to improve implementation. There is a need to include the document in the patient’s medical record. The reasons for which ADs have not been further developed are misinformation and culture (death is still perceived as a taboo). Any initiative on ADs must be made by the patient
Primary care (doctors and nurses)	2009	Valle, A Observational	113 participants. 68.1% were aware of the possibility of registering ADs. 70% believed that the initiative to talk about ADs had to be made by patients themselves. 53.2% considered the primary care visit to be the appropriate environment for addressing ADs. 60.7% would feel comfortable addressing the issue. The main difficulties in addressing it were: talking about death with patients (52.2%) and explaining administrative procedures (45.1%). 55.4% believed that the population would be interested in completing ADs
Primary care (doctors, nurses, assistants, social workers)	2010	Champer, Anna Observational	227 participants. 83.8% knew the definition of ADs. Only 4.1% knew about their legal aspects, 0.5% the registration procedure, 1.4% the document content and 38.6% the document’s purpose. Only 4 professionals out of 277 had prepared ADs
Primary care (doctors, nurses, social workers)	2015	Fajardo, MC Observational	340 participants. 78.4% believed that ADs were regulated by law. 33.9% of doctors, 36.4% of nurses, and 100% of social workers had read the document. Those surveyed were willing to prepare their own ADs and to use them, were aware of their utility and wanted them to be respected by health professionals
Primary care (doctors, nurses)	2015	Jiménez, José M Observational	85 participants. 95.3% knew that ADs were regulated by law, 40% had read them at some time, and 37.6% were familiar with the provincial AD Register. 24.7% had read the guide on ACP, and 12.9% had made plans in advance with patients about their final wishes during a doctor’s visit. Few professionals were truly knowledgable about the document, but it was considered a useful tool for clinical practice, requiring better training for professionals, and increased dissemination and awareness among the healthy population
Primary and specialized care (doctors and nurses)	2008	Simón Lorda, Pablo Observational	298 nurses: 63.1% knew that ADs were regulated by law, and 32.3% had read an AD document at some time. Most believed that it was advisable to plan and prepare the patient’s wishes regarding health care, considering ADs to be a useful tool for professionals and relatives. High willingness to complete ADs, though not in the very near future, was found
		Simón Lorda, Pablo Observational	194 doctors. 69.6% knew that ADs were regulated by law, and 37.6% had read an AD document at some time. Most believed that it was advisable to plan and prepare the patient’s wishes regarding health care, considering ADs to be a useful tool for professionals and relatives. High willingness to complete ADs, though not in the very near future, was found
	2013	Toro, Rafael Observational	192 participants. 60.1% knew about the legal regulation of ADs, above all primary care doctors and nurses. 22.8% had read ADs. A favorable attitude was found towards the use of, utility of and respect for ADs content. Primary care doctors and hospitalization nurses showed a more favorable attitude towards ACP than did hospital doctors and primary care nurses
Primary care and specialists (doctors)	2013	Ameneiros-Lago, Eugenia Observational	120 participants. 17.5% had detailed knowledge of ADs, with specialist doctors showing the best knowledge, and more than 10 years experience. 23.3% had at some point explained the advisability of preparing ADs to patients, with 6.7% helping them do so. 90.8% showed a positive attitude towards their usefulness and 87.9% would be willing to complete ADs
Primary care and specialists	2018	Martínez, ML Observational	431 participants. Lack of knowledge about both ADs and advanced decision planning. 4.6% had ADs, and 42% were unaware of regional regulation. Positive attitude towards the usefulness of the documents and considered it convenient to plan care with patients
Primary care and specialists (doctors and nurses)	2018	Aguilar, Juan M Observational	329 participants. Low level of knowledge, especially about document content, legal aspects and procedure. 18.5% had had experience handling them, and 22.2% had read an AD at some time. 94.5% would participate in training activities, showing a very positive attitude towards the document
Specialized care (doctors and nurses)	2011	Franco Tovar, Begoña Observational	607 participants. Only at one hospital out of eight was there a valid ADs protocol. 12% indicated that the preferences of terminally-ill patients were explored in their ward, especially in the ICU
Specialized care (doctors and nurses)	2014	Sepúlveda, Juana M Qualitative study	17 participants. Professionals felt uncomfortable asking and informing about ADs, although they considered it very important for the patient’s wishes to be respected. Nurses stated they had greater difficulty accessing the ADs register and content. They expressed the need to further both undergraduate and graduate training on how to approach terminal patients
Specialized care (doctors, nurses, assistants)	2016	Pérez, María Observational	283 participants. 84% never informed their patients about ADs. Reasons: 33.9% did not consider it a part of their job, 21.2% claimed they do not have enough time, and 18.3% did not know what ADs were. The patient profile they believed should receive information was: 77% terminally-ill patients, 61% chronically-ill patients and 43% the elderly. Regarding who should provide that information, 62.6% considered the primary care doctor to be the key player. 57% knew what ADs were, 19% how to complete them and 16% their legal regulation. 83% considered patient involvement in ADs completion to be important. 79% expressed their desire to complete ADs. The degree of knowledge was higher among the medical services compared with surgical services, and doctors compared with nurses
Specialized care (doctors, nurses)	2020	Herreros, Benjamín Qualitative study	60 participants. Many professionals considered ADs to be a bureaucratic procedure with no real impact on the quality of clinical practice. They showed a lack of professional awareness about the utility of ADs. They also considered it a complex procedure, causing it to be non-user friendly and hindering use. The information received by professionals on ADs was inadequate, and there were professionals who, having received training on ADs, did not use them
Specialized care (nurses)	2020	Vázquez, Miriam Observational	262 participants. 2% felt that they had enough information, 50% believed that professionals are required to provide information on ADs, and 13% said that patients are not well informed. From 61 to 93% fail to answer questions related to documentation, use and legal issues. 84% believe that respecting patients’ values and beliefs is mandatory and 89% that patients had the right to decide about care. Most would recommend that their chronic patients prepare such a document
Emergencies and ICU (doctors)	2010	Nebot, Cristina Observational	84 participants. 6% had consulted the ADs Register. Reasons for not consulting the register: 28% stated that they did not have a password or know how to consult it
Emergencies (doctors and nurses)	2007	Mateos, Alonso A Observational	49 participants. 73.5% claimed to be familiar with ADs and 18% with the current legislation on ADs. 51% had at some time asked whether anyone knew the patient’s preferences before beginning CPR maneuvers. 83.3% were willing to complete ADs, though none had done so
	2015	Dorribo, Marta Observational	71 participants. 85.9% knew what ADs were, 39.4% had at some time read ADs, but most did not know whether an Autonomous Community law governed them. 40.8% indicated that they can be consulted through the medical records. 84.5% knew that ADs foresee limits on medical action. The vast majority were unaware that the person’s values may also be reflected. 56.3% had not considered the possibility that a terminally ill patient had prepared ADs. Most considered them to be a useful document for patients’ relatives. None had prepared ADs, but 16.9% considered the possibility of doing so in the next year. Factors influencing poor implementation: patients’ biases towards these issues and little information from professionals
Intensive Care (doctors and nurses)	2016	Velasco, Tayra Observational	331 participants. 90.25% were unaware of the steps foreseen in ADs. 90.6% did not know whether the patients in their care had ADs. 50.2% indicated that ADs were not honored when required. 82.8% considered them to be a useful tool for professionals in decision-making
Residences (doctors, nurses, nurse aids, social worker, occupational therapist)	2017	Sánchez, María R Qualitative study	15 participants. Difficulties in communication with families, related to feelings of guilt, difficulty in understanding deterioration and approaching the subject of death late in the process. Other difficulties found were a lack of training, resources and coordination among the various professionals. They did not encourage patient participation in decision-making. They considered ADs a necessary tool, though they did not foresee their implementation in a systematic way
Mental Health (doctors, nurses, nurse aids, psychologists)	2019	Juliá-Sanchis, Rocío Qualitative study	11 participants. Importance of ADs in mental health. Preparing ADs on treatment preferences is an important opportunity for people with mental illness, in the event of hospitalization or temporary disabling events because, among other problems, it can help prevent conflict for family members and professionals. Some of the difficulties in ADs implementation included a lack of knowledge and barriers to their practical management, the fallacy of empowerment with latent paternalism, a paradoxical view of the role played by families, stigma … the importance of developing professional skills to implement ADs in mental health, determining who, what, where and how to address the issue

For all of these reasons, ADs can be said to have failed in Spain: because its prevalence is very low, there is a great lack of knowledge about them, ADs have almost no impact on clinical decisions and, therefore, they have not achieved their goal (to honor autonomous patient decisions about future care). This article aims to analyze and discuss the main reasons which have led to the failure of ADs in Spain, with the intention of seeking out strategies to improve their implementation into the future.

## Main text

According to the studies carried out in Spain, the main reasons why ADs do not have an impact on clinical practice have been: (1) deficient training of health professionals on the end of life and ADs; (2) the lack of a public process to increase awareness about ADs documents; (3) excessively cumbersome bureaucratic documentation and implementation procedures; (4) the continued existence of a paternalistic medical culture.

### Poor training of health care professionals

Health care professionals possess little knowledge about ADs. These studies were mainly carried out among doctors and nurses [12, 13], though there are also studies in which other types of health care professionals took part. Although the knowledge among these professionals generally reaches a level higher than among the general public, most of these professionals do not possess detailed knowledge about ADs (as a concept), the laws currently in force (including their binding nature in decision-making processes) and, above all, how ADs can be put into practice; this ranges from administrative aspects (they are usually unaware of how to consult the corresponding ADs Register) to the manner in which they are supposed to proceed in a specific clinical case [14, 15]. This lack of knowledge exists in all units [16, 17], including those in which patients are often subject to incapacitation (ICU, internal medicine wards, palliative care) [18, 19]. One result of this deficient training among health care professionals is that most have never informed their patients about ADs and do not, in general, know whether the patients in their care have filled out and registered ADs. All of this means that the previously stated wishes of patients expressed through ADs may be violated [20], even though the current Spanish legislation states that it is mandatory to consult whether the incapable patient has registered an AD and, in the case decisions have to be made and the patient do not have sufficient capacity to express their wishes or give informed consent, their wishes/preferences must be fulfilled.

When professionals are asked about their training, they acknowledge that it is lacking in terms of both the end of life (reporting bad news, the palliative care approach, coping with suffering, shared planning of care, grief) and ADs [21]. This is reflected by the fact that very few professionals have prepared their own ADs. Nevertheless, despite their lack of knowledge and training on ADs, most have a positive attitude towards them. They believe that ADs can be useful to both themselves and patients’ relatives, and therefore they are very much in favor of the increasing awareness about ADs and furthering their development [22], as well as improving their training. One statement repeated constanty over the years is that health care professionals demand greater training on ADs.

### Lack of a public awareness process

While it is essential for professionals to be trained on dealing with the end of life and, in particular, with ADs, for regular citizens it is essential to create a public process for increasing awareness about ADs documents. By doing this, they can become aware of what ADs are and how they can be documented and implemented. However, no effort has been made to disseminate and educate the public about ADs in Spain. This is reflected in the studies which have been completed; they show that the level of awareness among the public is even lower that that found among health care professionals. Regardless of the field of health care [23–25] and pathology [26–29], patients have proven to possess very little knowledge about ADs [30, 31]. Most have obtained information through the mainstream media, which can lead to confusion. Because this information is not mediated by health professionals, it can cause unfounded fears and prejudices, for instance by associating ADs with euthanasia or with doctors abandoning patients. In fact, in some Autonomous Communities, the person in charge to register the ADs with the citizen is not necessarily a healthcare professional, but a lawyer or administrative staff, as for example in the case of Andalusia.

The lack of a public awareness process means that most of the people who have prepared ADs tend to be individuals who are especially sensitive about end-of-life care because of their clinical or social characteristics, or their ideology. Most of the people who have drafted ADs in Spain [32, 33] are women; aged between 55 and 70 years; with an average to high level of education; independent in performing basic daily activities; and many suffer from a chronic pathology. It has been found that the patients and people who are most knowledgeable about ADs usually suffer from some chronic pathology. Chronic disorders cause people to reflect on the end of life, including both the patients themselves and those around them, and therefore the relatives of patients with chronic pathologies also possess greater knowledge about ADs. The same is true for critically ill patients: patients who have been admitted to the intensive care unit are more likely to participate in decision-making and to draft ADs, and having a family member or friend admitted to intensive care spurs reflection on ADs. It has also been found that many of the individuals who have registered an ADs document had a family member who had previously done so [34], that a significant number are the primary caretakers of patients at the end of life (usually women) and that others are activists in some private entity with clear wishes regarding the end of life, such as Jehovah’s Witnesses or advocates for the right to decide at the end of life.

After being given information, patients show a positive attitude towards ADs in general [35] and consider it important to offer everyone the chance to prepare them [36], as well as the need to include the document in the clinical record [37]. They believe that ADs would improve the relationship with health care professionals and give them peace of mind regarding future decisions made with them. It must not be forgotten that the main reasons for drafting ADs are to plan what interventions they do not want to have performed (limiting treatments such as life support) and to be able to receive drugs that relieve pain for fear of dying under poor conditions. In one study of dialysis patients, fewer than 15% had expressed their wishes about whether or not to continue with dialysis to their patient representative, and those who had done so had left no documented confirmation. After being informed about ADs, more than 6 out of 10 were willing to draft and register them [38].

Knowing that adequate awareness about the document has not been raised, patient believe that the key to its implementation is education for both them and health care professionals. There is now a greater culture of shared decision-making at the end of life, but much has yet to be done. There are still prejudices and taboos about how to face death [39], which makes it difficult to promote an open dialogue on the end of life among the ill, their relatives and health care professionals, thus affecting the completion of ADs.

There are also studies on patients’ relatives and representatives [40, 41], above all in intensive care units and emergency wards. They, too, show a great lack of knowledge about ADs documents and patients’ wishes, but show a positive attitude towards receiving information. In general, patient representatives (mostly women, aged 50–70 years, and usually spouses or daughters) believe that ADs are easy to use and practical, and that they give peace of mind to patients, as well as being helpful for receiving more respectful care when facing death, reaching consensus on decisions, honoring the decisions made by patients, serving to limit and prevent unwanted treatments, as well as preventing and shortening unnecessary suffering [42]. The opinion of the representatives that ADs are easy to use seems contradictory to the patients and, especially, health care professionals’ opinion. But it must be taken into account that the representatives do not carry out the AD registration process or their consultation (task of professionals). They are only consulted when necessary. Patient representatives also believe that greater efforts should be made to increase awareness about ADs.

### Excessively cumbersome bureaucratic procedure

The process for documentating and executing ADs in Spain is not easy for health care professionals or patients either, and that makes it difficult to make their use more widespread [43]. Usually, although it depends on the Autonomous Communities, the document cannot be accessed through the patient’s clinical record, and in order to consult the corresponding ADs Register, health care professionals must possess a set of personal passwords which expire periodically. As a result, if a case arises in which they must consult ADs, it is very likely that a doctor will not know how to consult the ADs Register (as stated above, due to a lack of training), and if they do know how to, their passwords are very likely to have expired. This has taken, for instance, in the Autonomous Community of Madrid. It is a cumbersome procedure which is not at all user-friendly for health care professionals.

In several studies, these professionals have pointed out that access to the ADs document should be made easier and that the ADs on record should be accessible through the patient’s clinical record. To encourage the use of ADs, it is important that the way in which they are documented and the system for consulting the ADs Register must be made simpler, with access provided through the patient’s clinical history, as is the case in Catalonia.

Another problem related to the bureaucratic procedure is that patients usually have to do ADs by themselves, without the help of a healthcare professional, therefore it is more difficult that patients register ADs. Furthermore, as previously noted, in some ADs registries, the person in charge is a lawyer or and administrative, but not a healthcare professional, therefore, many of the decisions can not be discussed appropriately with the person/patient.

In order to make the management and implementation of ADs easier to perform, professionals and patients believe that a priority role should be placed on primary care [44, 45]. Primary care professionals are more familiar with patients and can undertake dialogue with them in a better way in order to determine their health care priorities [46]. Patients think that doctors should inform them about ADs, especially their family physician. However, during acute processes or during specialist visits, it may also be appropriate to begin a dialogue about planning health care decisions and ADs. Another proposal of great interest for improving the way in which ADs work is to identify groups of patients on whom a priority should be placed, targeting those with terminal and chronic illnesses and the elderly [47]. Strategies for increased awareness, documentation and execution of ADs should begin with them.

### Paternalistic Spanish medical culture

Historically, Spanish and, in general, Mediterranean medicine has been characterized as communitarian and paternalistic. The patient’s cultural community (including their family and social environment) have been very present in the decision-making process, forming the basis of the doctor’s authority [48, 49]. This structure has existed in opposition to greater liberalism in the English-speaking world and Northern Europe, where patient autonomy in decision-making is more prevalent [50]. One of the aspects of bioethics with the greatest impact on Spanish medicine has been the introduction of respect for patient autonomy. ADs are a reflection of respect for patient autonomy (expressed in advance), and despite the fact that Spanish laws have consolidated this respect, studies carried out in Spain show that there are still remnants of medical paternalism among both health care professionals and the public.

One piece of data which shows how paternalism is still present in Spanish medicine, above all within the realm of hospitalization, is that most patients believe that having prepared ADs will not change the doctor’s attitude or decision [51, 52]. A study carried out in the field of psychiatry shows that there is a certain rejection of patient empowerment, with a latent paternalism persisting in decision-making and families playing a paradoxical role [53]. In a study of patient representatives carried out at Catalonia’s ADs Register [42], two out of three surveyed stated that the health care team read the ADs and usually believed that the patient’s will was honored, but only 59% said that the health care professionals asked for their opinion as patient representatives. It cannot be ignored that the main difficulty which this reflects is a potential mismatch between the health care professional’s criteria and the wishes expressed in the document.

As for health care professionals, they sometimes fail to facilitate patients’ decision-making either [54], which may be an example of what we have referred to as “latent paternalism.” For different reasons (lack of knowledge or time, failure to bear in mind that completing ADs is part of their work) [55], doctors almost never provide information on ADs and seldom help patients complete them [56]. Also among the problems which ADs [47] can create are discrepancies between the patient’s instructions and the opinion of family members or doctors, which may not be in line with proper clinical practice and may pave the way towards legal action. All of the aforementioned can make doctors feel defensive, as they are usually the ones who prefer to guide decision-making among patients.

Last of all, it should be pointed out that many patients trust their families and doctors more than they trust documents, a factor that cannot be blamed on medical paternalism alone. It has been found that patients usually prefer human relations to documents, which leads to the need to establish better-flowing communication and shared planning of decisions [57], instead of focusing decision-making on a mere document [58].

## Conclusions

Although there has been a specific legislative framework governing ADs in Spain for 20 years, and bioethics has developed in recent decades, the implementation of ADs has failed. ADs have not fulfilled their purpose, because they have hardly any impact on clinical decisions, and therefore they have not been a useful tool to help honor patients’ autonomous decisions about their future care. Studies indicate that there are four reasons for this failure: (1) the lack of proper training for health care professionals on the end of life and ADs, in terms of the conceptual framework, existing legislation and legal implementation; (2) lack of a public process to increase awareness about ADs documents, which has led to a great lack of knowledge about ADs among patients, and thus only certain groups especially sensitive to end-of-life issues (chronic and terminally ill patients, as well as their families, caregivers and certain ideological groups) are fully aware of them and register ADs properly; (3) excessively cumbersome bureaucratic documentation and implementation procedures, which are a barrier for patients to prepare them and for professionals to consult them when necessary; (4) the continued existence of a paternalistic medical culture, both among patients and health care professionals, which makes it difficult to reach shared decisions with patients and their relatives.

Despite the difficulties that have been identified, it has also been observed that health care professionals and the Spanish public have a very positive view of ADs. They believe that ADs can be very useful, insisting upon the importance of increasing awareness about ADs and very willing to receive information. Due to all of the above, once the problems that have kept ADs from becoming successful are identified, strategies must be developed to help further their implementation into the future. These strategies should include the development of Advance Care Planning (ACP). ACP is a structured approach that allows patients, relatives and physicians to discuss end-of-life decisions [59]. ACP enables individuals to define goals and preferences for future medical treatment and care, to discuss these goals and preferences with family and health-care providers, and to record and review these preferences if appropriate. The current restriction of ADs to the writing and signing of a document—in contrast to the discussion and review involved in ACP—has extensive limitations, such as a general lack of public trust in the documents, as evidenced by low completion rates. A new approach, involving the implementation of ACP, may be able to overcome some of these limitations.

## Data Availability

Not applicable.

## Abbreviations

ADs: Advance Directives
ICU: Intensive care unit
ACP: Advance Care Planning

## References

[1] Martínez León M, Queipo Burón D, Martínez León C, Justel Gómez E. Análisis médico-legal de las instrucciones previas (Living Will) en España. Rev Med Legal. 2008;16–30.

[2] Zabala Blanco J, Díaz Ruiz JF (2010). Reflexión sobre el desarrollo y utilidad de las instrucciones previas. Semergen.

[3] Spanish Register of Advanced Directives. https://www.mscbs.gob.es/ciudadanos/rnip/doc/Documentos-2020/Declarantes_con_Instruccion_Previa_Activa_por_Comunidad_Autonoma_y_grupos_de_edad_Enero_2020.pdf. Accessed 14 Mar 2020.

[4] Gracia Guillén D (2009). Spanish bioethics comes into maturity: personal reflections. Camb Q Healthc Ethics.

[5] Antolín A, Sánchez M, Llorens P, Martín Sánchez FJ, González Armengol JJ, Ituño JP (2010). Knowledge about disease course and living wills among patients with hearth failure. Revista Española de Cardiología.

[6] Monzón JL, Saralegui I, Camposc RAC, Cabré L, Iribarren S, Martín Delgado MC (2008). Recomendaciones de tratamiento al final de la vida del paciente crítico. Med Intensiva.

[7] Arauzo V, Trenado J, Busqueta G, Quintana S (2010). Grado de conocimiento sobre la ley de voluntades anticipadas entre los familiares y los pacientes ingresados en un servicio de medicina intensiva. Med Clin (Barc).

[8] Simón Lorda P, Tamayo Velázquez MI, Vázquez Vicente A, Duran Hoyos A, Pena Gonzales J, Jiménez ZP (2008). Conocimientos y actitudes de los médicos en dos áreas sanitarias sobre las voluntades vitales anticipadas. Aten Primaria.

[9] Mateos Rodríguez AA, Huerta Arroyo A, Benito Vellisca MA (2007). Instrucciones previas: actitud de los profesionales de emergencia. Emergencias.

[10] Contreras Fernández E, Barón López FJ, Méndez Martínez C, Canca Sánchez JC, Cabezón Rodríguez I, Rivas RF (2017). Validación del cuestionario de conocimiento y actitudes de los profesionales sanitarios en el proceso de declaración de voluntades anticipadas. Aten Primaria.

[11] Champer Blasco A, Caritg Monfort F, Marquet PR (2010). Conocimiento y actitudes de los profesionales de los equipos de atención primaria sobre el documento de voluntades anticipadas. Aten Primaria.

[12] Dorribo Masid M, Rodríguez Barreiro S, Gándara Quintanas CM, Sanz Smith FJ, López Álvarez XM, Rodríguez RA (2015). Conocimiento de los documentos de instrucciones previas en el servicio de emergencias de Galicia. Cad Aten Primaria.

[13] Velasco Sanz TR, Rayon VE (2016). Advance directives in intensive care: health professional competences. Med Intensive.

[14] Fajardo Contreras MC, Valverde Bolivar FJ, Jiménez Rodríguez JM, Gómezcalero A, Huertas Hernández F (2015). Grado de conocimiento y actitudes de los profesionales ante el documento de voluntades anticipadas: diferencias entre distintos profesionales y provincias de una misma autonomía. Semergen.

[15] Jiménez Rodríguez JM, Farouk AM (2015). Conocimiento, actitud y planificación de la voluntad vital anticipada en el Distrito Sanitario Guadalquivir de la provincia de Córdoba. Med Gen y Fam.

[16] Simón Lorda P, Tamayo Velázquez MI, González Rubio MJ, Ruíz Díaz P, Moreno González J, Rodríguez González MC (2008). Conocimientos y actitudes del personal de enfermería acerca de las voluntades anticipadas en 2 áreas sanitarias de Andalucía. Enferm Clín.

[17] Toro Flores R, Silva Mato A, Piga Rivero A, Alfonso Galán MT (2013). Conocimiento y actitudes de médicos y enfermeras sobre las instrucciones previas. Aten Primaria.

[18] Franco Tovar B, Da Silva Gamma ZA, Saturno Hernández PJ (2011). Conocimiento de las preferencias de los pacientes terminales en los hospitales públicos de la Región de Murcia. Rev Calid Asist.

[19] Sepúlveda Sánchez JM, Morales Asencio JM, Morales Gil IM, Canca Sánchez JC, Crespillo García E, Timonet Andreu EM (2014). El derecho a morir con dignidad en un hospital de agudos: un estudio cualitativo. Enferm Clin.

[20] Vázquez-Campo M, Tizón-Bouza E, Martínez-Santos AE, Vilanova-Trillo L (2020). Qué conocen las enfermeras de Galicia sobre las voluntades anticipadas. Enferm Clin.

[21] Martínez Gimeno ML, Cámara Escribano C, Honrubia Fernández T, Olmo García MC, Tovar Benito DH, Bilbao-Goyoaga Arenas T (2018). Conocimientos y actitudes sobre voluntades anticipadas en profesionales sanitarios. J Healthc Qual Res.

[22] Aguilar-Sánchez JM, Cabañero-Martínez MJ, Puerta Fernández F, Ladios-Martín M, Fernández-de-Maya J, Cabrero-García J (2018). Grado de conocimiento y actitudes de los profesionales sanitarios sobre el documento de voluntades anticipadas. Gac Sanit.

[23] Santos de Unamuno C (2003). Documento de voluntades anticipadas: actitud de los pacientes de atención primaria. Aten Primaria.

[24] Martínez Almazán E, Altadill Ardit A, García Navarro JA (2002). Disposiciones previas: experiencia piloto en una residencia de ancianos. Rev Esp Geriatr Gerontol.

[25] López Rey A, Romero Cano M, Tébar Morales JP, Mora García C, Fernández RO (2008). Conocimiento y actitudes de la población ante el documento de voluntades anticipadas. Enferm Clin.

[26] Domínguez Lorenzo C, García Verde I, Alonso Pérez MA, Muelas GS (2011). Conocimiento y utilización del documento de instrucciones previas por los pacientes en programa de cuidados paliativos. Med Paliat.

[27] Miró G, Pedrol E, Soler A, Serra-Prat M, Yébenes JC, Martínez R, Capdevila JA (2006). Conocimiento de la enfermedad y de los documentos de voluntades anticipadas en el paciente seropositivo para el VIH. Med Clin (Barc).

[28] Antolin A, Sánchez M, Llorens P, Martín Sánchez FJ, González Armengol J, Ituño JO (2010). Conocimiento de la enfermedad y del testamento vital en pacientes con insuficiencia cardiaca. Rev Esp Cardiol.

[29] Sánchez Tomero JA, Rodríguez Jornet A, Balda S, Cigarrán S, Herrero JC, Maudell F (2011). Evaluación de la opinión de los pacientes con enfermedad renal crónica en diálisis respecto al fin de la vida y la planificación anticipada de cuidados. Nefrologia.

[30] Angora Mazuecos F (2008). Voluntades Anticipadas vs Instrucciones Previas o Testamento Vital en Atención Primaria. Rev Clin Med Fam.

[31] Serrano Teruel R, López López R, Illana Rodríguez J, Alfonso Cano C, Sánchez López MI, Leal Hernández M (2015). Documento de instrucciones previas. ¿Conocido por nuestros pacientes?. Educ Med.

[32] Antolín A, Jiménez S, González M, Gómez E, Sánchez M, Miró O (2014). Características y uso del documento de voluntades anticipadas en un hospital terciario. Rev Clin Esp.

[33] Nebot C, Ortega B, Mirab JJ, Ortiz L (2010). Morir con dignidad, Estudio sobre voluntades anticipadas. Gac Sanit.

[34] Del Pozo K, López Torres J, Simarro MJ, Navarro B, Rabanales J, Gil V (2014). Características sociosanitarias de quienes formalizan el documento de voluntades anticipadas. Semergen.

[35] Llordés Llordés M, Zurdo Muñoz E, Serra Morera I, Giménez GN (2014). Conocimientos, expectativas y preferencias respecto al documento de voluntades anticipadas entre los pacientes de atención primaria. Med Clínica.

[36] Andrés-Pretel F, Navarro B, Párraga I, de la Torre MA, Jiménez MD, López-Torres J (2012). Conocimientos y actitudes de los mayores hacia el documento de voluntades anticipadas. Gac Sanit.

[37] Flordelís MF (2000). Testamento vital en las historias clínicas. Trabajo Social y Salud.

[38] Rodríguez Jornet A, Betancourt Castellanos LA, Bolós Contador MA, Oliva Morera JC, Ibeas López JA (2017). Usefulness of questionnaires on advance directives in haemodialysis units. Nephrol Dial Transplant.

[39] Ortiz-Gonçalves B, Santiago-Sáez A, Albarrán Juan E, Labajo González E, Perea-Pérez B (2018). Elaboración de un cuestionario sobre conocimientos y actitudes de la población madrileña frente al final de la vida. Gac Sanit.

[40] Solsona Durán JF (2007). Voluntades anticipadas: una herramienta para anticipar acontecimientos y facilitar la asistencia urgente. Emergencias.

[41] Antolin A, Ambrós A, Mangirón P, Alves D, Sánchez M, Miró O (2010). Grado de conocimiento del documento de voluntades anticipadas por el enfermo crónico que acude a urgencias. Rev Clin Esp.

[42] Busquets JM, Hernando P, Font R (2016). Los documentos de voluntades anticipadas La opinión de los representantes. Rev Calid Asist.

[43] Sánchez-García MA, Moreno-Rodríguez M, Hueso-Montoro C, Campos-Calderón C, Varella-Safont A, Montoya-Juárez R (2017). Dificultades y factores favorables para la atención al final de la vida en residencias de ancianos: un estudio con grupos focales. Aten Primaria.

[44] Navarro Bravo B, Sánchez García M, Andrés Pretel F, Júarez Casalengua I, Cerda Díaz R, Párraga Martínez I (2011). Declaración de voluntades anticipadas: estudio cualitativo en personas mayoras y médicos de Atención Primaria. Aten Primaria.

[45] Lauzirika N (2007). Los médicos de atención primaria, eslabón clave en los testamentos vitales. El médico.

[46] Valle Sánchez A, Farrais Villalba S, González Romero PM, Galindo Barragán S, Rufino Delgado MT, Marco García MT (2009). Documento de voluntades anticipadas: opinión de los profesionales sanitarios de Atención Primaria. Semergen.

[47] Herreros B, Monforte MJ, Molina J, Velasco M, Olaciregui Dague K, Valenti E (2020). The use of advance directives in specialized care units: a focus group study with healthcare professionals in Madrid. J Bioeth Inequal.

[48] Busquets E, Roman B, Terribas N (2012). Bioethics in Mediterranean culture: the Spanish experience. Med Health Care Philos.

[49] Gracia GD (1995). A Mediterranean model of bioethics?. An R Acad Nac Med (Madr).

[50] Gracia D (1993). The intellectual basis of bioethics in Southern European countries. Bioethics.

[51] Molina J, Pérez M, Herreros B, Martín MD, Velasco M (2011). Conocimiento y actitudes ante las instrucciones previas entre los pacientes de un hospital público de la Comunidad de Madrid. Rev Clin Esp.

[52] Pérez M, Herreros B, Martín MD, Molina J, Guijarro C, Velasco M (2013). Evolución del conocimiento y de la realización de instrucciones previas en los pacientes ingresados en medicina interna. Rev Calid Asist.

[53] Juliá-Sanchis R, García-Sanjuan S, Zaragoza-Martí MF, Cabañero-Martínez MJ (2019). Advance healthcare directives in mental health: a qualitative analysis from a Spanish healthcare professional’s viewpoint. J Psychiatr Ment Health Nurs.

[54] Santos C, Forn MA, Pérez R, Corrales A, Ugarriza L, Sales C (2007). ¿Estamos preparados los médicos de familia para ayudar a nuestros pacientes a hacer El testamento vital?. Rev Calid Asist.

[55] Pérez M, Herreros B, Martín MD, Molina J, Kanouzi J, Velasco M (2016). Do Spanish hospital professionals educate their patients about advance directives? A descriptive study in a University Hospital in Madrid, Spain. J Bioeth Inqual.

[56] Ameneiros-Lago E, Carballada-Rico C, Garrido-Sanjuán JA (2013). Conocimientos y actitudes sobre las instrucciones previas de los médicos de Atención Primaria y Especializada del área sanitaria de Ferrol. Rev Calid Asist.

[57] Palero Castelló C. Documento de instrucciones previas y afrontamiento del proceso de morir en el enfermo renal crónico terminal en hemodiálisis. Perspectiva antropológica. Seden. 2009;Suppl. XXXIV National Congress SEDEN:36–40.

[58] Saralegui I, Lasmarías C, Júdez J, Pérez de Lucas N, Fernández J, Velasco T, Limón Ramírez E, Meléndez Gracia A (2018). Claves en la planificación compartida de la atención Del diálogo al documento. Monografía SECPAL sobre Cronicidad Avanzada.

[59] Rietjens JAC, Sudore RL, Connolly M, van Delden JJ, Drickamer MA, Droger M (2017). Definition and recommendations for advance care planning: an international consensus supported by the European Association for Palliative Care. Lancet Oncol.

